# A Prosperous Application of Hydrogels With Extracellular Vesicles Release for Traumatic Brain Injury

**DOI:** 10.3389/fneur.2022.908468

**Published:** 2022-06-02

**Authors:** Yang Chen, Jingquan Lin, Wei Yan

**Affiliations:** Department of Neurosurgery, The Second Affiliated Hospital, Zhejiang University School of Medicine, Hangzhou, China

**Keywords:** hydrogels, exosomes, traumatic brain injury, biocompatible materials, therapy

## Abstract

Traumatic brain injury (TBI) is one of the leading causes of disability worldwide, becoming a heavy burden to the family and society. However, the complexity of the brain and the existence of blood-brain barrier (BBB) do limit most therapeutics effects through simple intravascular injection. Hence, an effective therapy promoting neurological recovery is urgently required. Although limited spontaneous recovery of function post-TBI does occur, increasing evidence indicates that exosomes derived from stem cells promote these endogenous processes. The advantages of hydrogels for transporting drugs and stem cells to target injured sites have been discussed in multitudinous studies. Therefore, the combined employment of hydrogels and exosomes for TBI is worthy of further study. Herein, we review current research associated with the application of hydrogels and exosomes for TBI. We also discuss the possibilities and advantages of exosomes and hydrogels co-therapies after TBI.

## Introduction

Every year, more than fifty million individuals worldwide suffer from traumatic brain injury (TBI). One in two people will experience one or more brain injuries of varying degrees in their lifetime (1). Many survivors of TBI left distinct types of permanent neurological deficits, which affect their ability to take care of themselves in their daily lives. TBI brings a heavy economic burden to the family and society. There are constantly new therapeutics verified in preclinical trials. However, no current medical intervention explicitly improves the prognosis of TBI in both preclinical and clinical trials (2). Traditional therapies only aim at several mechanisms of TBI. More importantly, inefficiency and side effects limit the development and use of these treatments.

Nevertheless, the emergence and rapid development of emerging treatment methods in recent years may break the current dilemma. The extracellular matrix (ECM) is a substance with a specific structure and function existing in all tissues and organs. It provides physical support for cells and transduces signals associated with cell proliferation, adhesion, and migration (3). Hydrogels can simulate ECM, and more vitally, can synergize with drugs or cell therapies. At present, the advantages of hydrogels for the treatment of TBI were prominent in many preclinical trials. The development and application of this new material have brought new hope for TBI treatment. Additionally, exosomes (a kind of vesicles with membrane structure) have been proved to play a vital role in intercellular communication. Therefore, research on exosome therapy in TBI also has become a hot topic in recent years.

In this review, we discuss recent advances that describe the basic properties of hydrogels and consider the use of hydrogels for the treatment of TBI and for amplification of effects of drugs and cell therapies to improve neurological recovery after TBI. Moreover, we discuss evidence of how unmodified or modified exosomes affect neurological outcomes when used as therapy for TBI. At last, we prospect opportunities for and challenges in a combined hydrogel-based exosome therapy to applications for TBI. It is worth noting that, as a part of the central nervous system, the situation of spinal cord injury (SCI) is similar to TBI. Therefore, in the review of the application of hydrogels and exosomes, we also include research on the application in SCI.

## Pathophysiology of TBI

**Figure 1:**
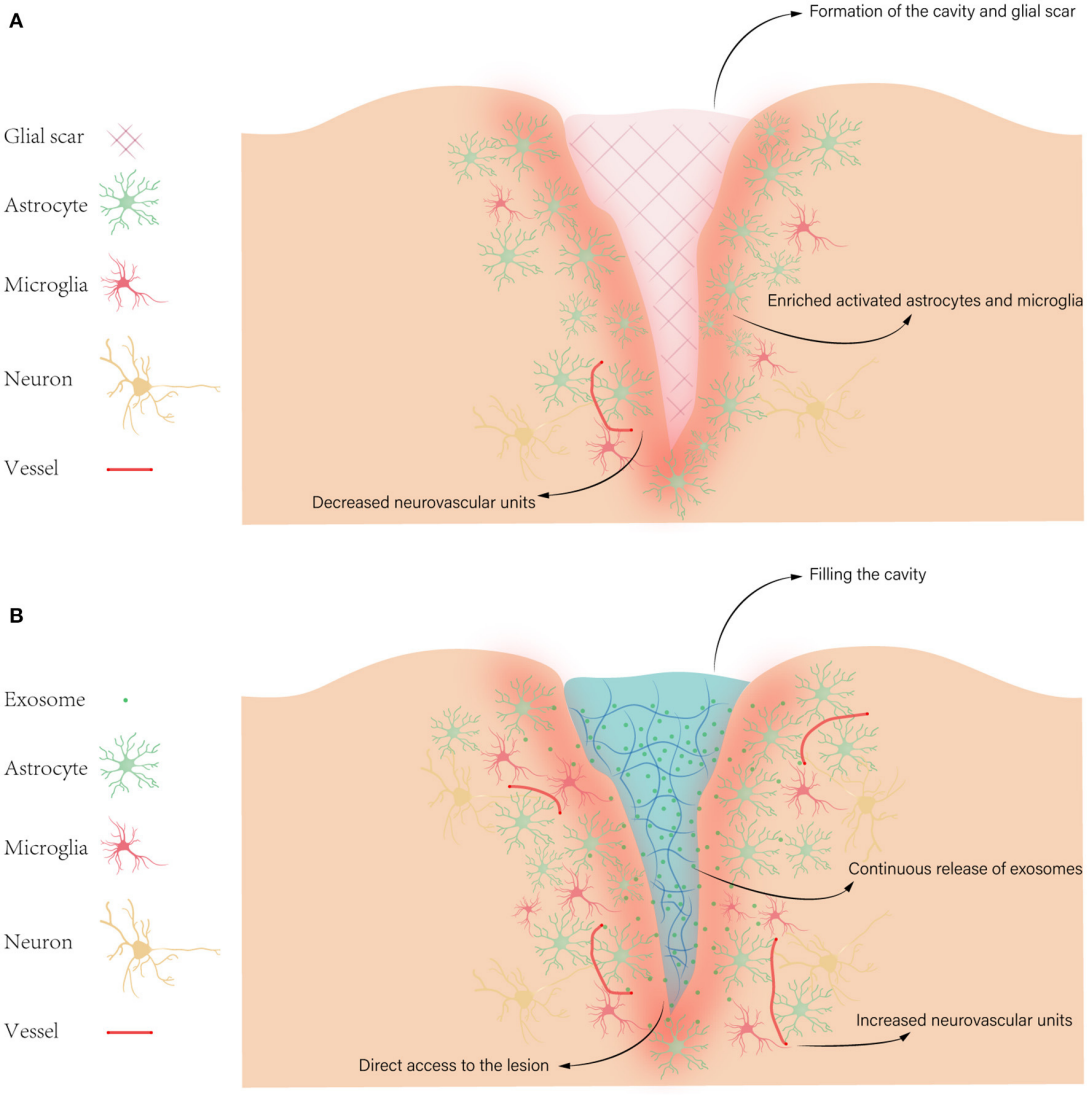
Pathophysiology of TBI. **(A)** Some pathophysiology of secondary injury after TBI, such as formation of the cavity and glial scar, enriched activated astrocytes and microglia, and decreased neurovascular units. **(B)** The possible advantages of hydrogels for exosome treatment in TBI, like filling the cavity, direct access to the lesion, increased neurovascular units, and continuous release of exosomes. Then exosomes target various cellular and molecular pathophysiological states, such as excitotoxicity, oxidative stress, neuroinflammation, and apoptosis.

According to damages of neuronal tissues, TBI can be divided into two main categories: primary injury and secondary injury. Primary injury occurs within seconds to minutes due to direct mechanical forces. The primary injury could lead to hemorrhages such as epidural hematoma, subdural hematoma, subarachnoid hemorrhage, intraventricular hemorrhage; focal cerebral contusions; traumatic axonal injury caused by shearing of white matter tracts; and cerebral edema. The secondary injury occurs after initial injury, characterized by the expansion of damage from the center of the trauma (4). Given the irreversible tissue damage and cell death caused by primary injury, we only focus on the mechanism of secondary injury. So far, numerous factors in secondary injury have been investigated, such as excitotoxicity, oxidative stress, neuroinflammation, apoptosis, axonal degeneration, and formation of cavity and glial scar (5) (Figure 1).

## Hydrogels

Hydrogels are cross-linked macromolecular networks that contain a large amount of water, making these materials friendly to water-enriched biological environments (6). Early work on hydrogels can be traced back to the mid-1930s (7). Due to high moisture content and potentials for biomedical applications, hydrogels have received a great deal of attention in recent years. Hydrogels are held together in many ways to form polymeric structures, including chemical bonds and intermolecular forces. Natural polymers such as hyaluronic acid (HA), gelatin, alginates, chitosan, pectin, collagen, and fibrin can be directly employed to prepare hydrogels. However, potential problems like immunogenicity and histocompatibility may limit the applications of natural hydrogels. Additionally, compared with natural hydrogels, synthetic hydrogels have better controllability, histocompatibility, and immunogenicity (8). Therefore, recent articles mainly report synthetic hydrogels.

## Structure and Mechanical Properties of Hydrogels

Before preparing a hydrogel, the structure and mechanical properties of the hydrogel should be considered, such as strength, stiffness, mesh size, and porosity. The effects of hydrogels are closely related to their characteristics, such as cytocompatibility, histocompatibility, and the release of therapeutic agents. For instance, the mechanical properties affect the viability and function of encapsulated cells, associating with the efficiency of cell therapy (9–16).

The mechanical strength and stiffness of hydrogels are measured by the compressive modulus directly determined by the cross-link density. And the percent composition of monomers, the polymer molecular mass, and the total amount of cross-linker are closely related to the hydrogels' cross-link density. Therefore, we can adjust the compressive modulus by changing these properties (17–22). The mechanical strength of hydrogels should match the target tissue. As for brain tissue, the ideal hydrogel is soft. In other words, the brain cell prefers the hydrogel with a low compressive modulus (≤3.8 kPa) (15). Many studies have revealed that neurons tend to grow on substrates with softer surfaces (0.1–1.0 kPa), while astrocytes and oligodendrocytes prefer stiffer compressive moduli (0.5–10 kPa) (10, 11, 23–28). These studies demonstrate that the mechanical characteristic of hydrogels plays a vital role in the proliferation and growth of brain cells. Due to the degradation and the loss of cross-links, the compressive modulus of hydrogels will gradually decrease, leading to the destruction of integrity (19, 29, 30). It is rare to see adult brain tissue suffering stretching or shearing force unless under extreme conditions. Thus, we believe that the compressive modulus deserves more attention in the mechanical properties of hydrogel when applied to TBI. As some studies point out that, mild TBI, neuroinflammation, hypertension, and even neural cell migration may cause a rise in the local or overall pressure of brain tissue (31–33).

Mesh size is defined as the distance between the cross-link points in the hydrogel, which is measured in angstroms (Å), usually from 10 to 150Å (8). It is a nanoscopic physical characteristic of the hydrogel, contributing to mechanical properties like stiffness. Given the need for exchanging nutrients and wastes with the surrounding environment, mesh size is a vital agent to consider when designing hydrogels. Moreover, diffusion rates are closely related to the size of the molecule and the mesh, determining the exchange efficiency of fluids and small molecules. By contrast, the migration of larger molecules and cells is affected by the degradation of hydrogels (34, 35).

The function of hydrogels also relates to macroscopic architecture, like interconnected pores or the general shape and size. Some cells and tissues serve functions based on large spaces within hydrogels. Therefore, the pore size is employed to measure the interior space of hydrogels. The formation of the pore can be natural or intentional. Many studies have successfully created varied sizes of pores by various complex methods (36–42). The size of interconnected pores in hydrogels regulates cell-specific growth and differentiation. Two studies prove the neurite outgrowth promoted before hydrogels degradation (41, 43). This characteristic can be deployed in the reconstruction of neural circuits. The general shape should be considered first when designing a hydrogel. Before gelation, most hydrogels will form any shape desired because of their mobility. To reconstruct a neural circuit, the hydrogel can be made as a strand to link two remote regions. The size of hydrogels must be considered due to the limited intracranial space. The swelling of hydrogels mentioned in many studies may lead to a sustained increase in size. But Lampe et al. reported the restrained swelling of hydrogels induced by the counterforce from surrounding brain tissue. And degradation of hydrogels also slowed down the increase in size caused by swelling (44).

## Application of Hydrogels in TBI

We review the literature on applications of hydrogels in TBI treatment and divide them into three categories: 1. Hydrogel alone; 2. Hydrogel as a drug delivery tool; 3. Hydrogel as a cell therapy delivery tool. Due to the limited effect, there are few reports about using hydrogel alone. Zhang et al. injected a hydrogel form of urinary bladder matrix (UBM) derived from porcine bladder tissue into the brains of TBI rats. They observed the good biocompatibility of UBM within the brain tissue, the reduced volume of the lesion and the relief of myelin disruption, improving vestibulomotor function (45). Similarly, Yun et al. prepared a hydrogel form of ECM from normal porcine brain tissue to inject into the brains of TBI mice, reducing lesion volume and improving neuro-behavioral function (3). As the focus of this review, we describe the related research of hydrogels in drug and cell therapies in the following part.

## Drug Delivery With Hydrogels

As excellent means of transportation with controllable degradation behavior, hydrogels are suitable tools to deliver drugs, playing significant roles at the target site within a specific time according to requirements. Based on secondary injury mechanisms post-TBI, factors such as oxidative stress and neuroinflammation can persist for a long time. Therefore, many studies on drug delivery with hydrogels in TBI use antioxidant or anti-inflammatory drugs (Table 1). Qian et al. developed an injectable TM/PC hydrogel composed of triglycerol monostearate (TM), hydrophobic poly (propylene sulfide) 120 (PPS120) and curcumin. They demonstrated that the TM/PC (PPS120 and curcumin) hydrogel responded effective immediately to the microenvironment after TBI, slowly releasing the drug up to 14 days and significantly reducing the ROS (46). The high porosity of hydrogels provides resident space for molecules and permits the release of molecules from the hydrogels' network at different rates. Thus, the release profile of the molecule can be modulated by mesh size changed by the crosslinking density of hydrogels. Some researchers take advantage of this feature to achieve the sustained release of ferulic acid, significantly inhibiting the oxidative stress enhanced in the early stage after TBI (47).

**Table 1:** Drug delivery with hydrogels in TBI or SCI.

**References**	**Disease**	**Model**	**Drug**	**Hydrogel base**	**Characteristic of hydrogel**
Qian et al. (46)	TBI	PBI, mouse	Curcumin	TM and PPS^a^_120_	• Injectable • Self-assembled • MMPs- and ROS-responsive^b^
Jeong et al. (48)	TBI	CCI, rat	HA-DXM^c^	PEG-bis-AA^d^	• Implantable • Cross-link in response to ultraviolet • Hyaluronidase/esterase affects amount of dexamethasone released
He et al. (49)	SCI	Aneurysm clip compression, rat	KAFAK/BDNF^e^	HAMC^f^	• Injectable • Fast gelling at physiologic temperatures minimal • Swelling properties
Maclean et al. (50)	TBI	PBI, mouse	Fucoidan	Fmoc-DIKVAV^g^	• Injectable • Self-assembled • Cross-link in response to ultraviolet • Promoting neurite outgrowth • Similar modulus with various region of brain
Dong et al. (47)	TBI	N/A	Ferulic acid	Chitosan/gelatin/β-glycerol phosphate	• Injectable • Thermosensitive

a *Triglycerol monostearate and hydrophobic poly (propylene sulfide)_120_*;

b *matrix metalloproteinases- and reactive oxygen species- responsive*;

c *Dexamethasone-conjugated hyaluronic acid*;

d *poly (ethylene glycol)-bis-(acryloyloxy acetate)*;

e *KAFAK, Amino acid sequences; BDNF, brain-derived neurotrophic factor*;

f *hyaluronan-methylcellulose*;

g *a laminin-inspired peptide sequence*.

Compared to intravascular administration, the injectable hydrogels bypass the BBB and direct contact with the injured site. Moreover, the release rate of drugs in hydrogels can be controlled. These characteristics of hydrogels may reduce the amount of drug-using and avoid the occurrence of side effects. It has advantages in the treatment of diseases like TBI where damage factors exist for a prolonged period. Jeong et al. placed dexamethasone in a hydrogel constructed by PEG-bis-AA/HA [photo-cross-linkable poly (ethylene) glycol-bis-(acryloyloxy acetate)/hyaluronic acid], demonstrating that compared with traditional intraperitoneal injection, this new drug delivery strategy markedly reduced the dosage of dexamethasone, as much as half above (48).

The formation of the cystic cavity post-TBI is common. This unfavorable environment is one of the factors inhibiting nerve regeneration. As a cavity implant with great plasticity, the hydrogel can perfectly fill the capsule cavity owing to its physical properties and create a favorable microenvironment beneficial to nerve regeneration and functional recovery post-TBI. He et al. have similar findings in the rat model of spinal cord injury (SCI). As a drug delivery platform, the brain-derived neurotrophic factor-modified hyaluronan-methylcellulose (HAMC) hydrogel slowly released anti-inflammatory peptides and nerve growth factors, which inhibited post-traumatic neuroinflammation and promoted axon regeneration in a longer time (8 weeks) (49). Although the formation of glial scars dominated by reactive astrocytes is essential for limiting the spread of inflammation, it is thought to hinder the regeneration of axons after trauma. Maclean FL et al. have developed a new self-assembling peptide hydrogel carrying anti-inflammatory macromolecule fucoidan. Compared with the untreated group, this hydrogel markedly reduced glial scar formation. On the one hand, the hydrogel filled the cyst left after trauma and supported the surrounding brain tissue, avoiding further damage caused by the collapse of the injured area. On the other hand, the presence of fucoidan reduced reactive astrocytes production (50).

## Tissue Engineering Scaffolds From Hydrogels

Stem cell therapy is one of the options in TBI therapies. However, the matrix environment caused by the secondary injury post-TBI is not conducive to the growth and proliferation of stem cells, greatly affecting their survival after transplantation. Further, this harmful environment may induce stem cells, such as endogenous neuro stem cells, to differentiate into astrocytes instead of neurons (51, 52). The possible immunogenicity of transplanted stem cells also limits the application. However, at present, the application of injectable hydrogels in TBI has brought dawn to stem cell therapy. Injectable hydrogels provide a favorable niche for enhancing stem cell therapy by improving the survival rate of stem cells (53). We summarize the application of hydrogels as stem cell scaffolds in TBI (Table 2).

**Table 2:** Tissue engineering scaffolds from hydrogels in TBI.

**References**	**Model**	**Cell**	**Drugs**	**Hydrogel base**	**Characteristic of hydrogel**
Ma et al. (53)	CCI, rat	BMSCs^a^	SDF-1^b^	SA/Col^c^	• Injectable • Fast gelling (7 min) • High water content (>97%) low hemolysis rate (<1%) • Good cytocompatibility
Alvarado-Velez et al. (54)	CCI, rat	BMSCs	FasL	Agarose	• Injectable • Cross-link in response to temperature • Reducing the cytotoxic CD8^+^ T cell population at the transplantation site
Zheng et al. (55)	Cryogenic, rat	hAMSCs^d^	SDF-1α	GelMA-imid^e^	• Injectable • Cross-link in response to blue light (405 nm) • Low module (95 Pa) • High compression stress (5.2 kPa) • Promoting the migration and differentiation of hAMSCs
Yao et al. (56)	CCI, rat	BMSCs	GOX/HRP^f^	GH^g^	• Injectable • High water content (>90%) • Similar storage modulus with brain • GOX regulates gelling time and the enzymatic degradation rate
Zhang et al. (57)	CCI, rat	hUC-MSCs^h^	N/A	HA/SA^i^	• Injectable • Fast gelling (6 min) • High water content • Appropriate rheological behavior • Longer degradation time
Jahanbazi Jahan-Abad et al. (58)	CCI, rat	hNS/PCs^j^ and hADSCs^k^	N/A	PuraMatrix	• Injectable • Promoting cell incorporation, migration, and proliferation • Resistance to proteolytic digestion • High insolubility • Apparent lack of cytotoxicity
Xu et al. (59)	PBI, rat	NSPCs^l^	Sema 3A^m^	Matrigel	• Implantable • Longer degradation time • Promoting cell migration and proliferation
Betancur et al. (60)	CCI, rat	NSCs^n^	N/A	CS-GAG^o^	• Injectable • Cross-link in response to ultraviolet • Promoting FGF2^n^ retention and maintaining the undifferentiated state of NSCs
Shi et al. (61)	PBI, rat	hUC-MSCs and astrocytes	BDNF^p^ and CXCR4^q^	RADA16^r^	• Injectable • Self-assemble • LOW cytotoxicity • Excellent cytocompatibility
Xue et al. (62)	CCI, rat	NSCs and EPCs^s^	N/A	PuraMatrix	• Injectable • Promoting cell incorporation, migration, and proliferation • Resistance to proteolytic digestion • High insolubility apparent • Lack of cytotoxicity

a *Bone marrow mesenchymal stem cell*;

b *stromal cell-derived factor-1*;

c *sodium alginate/collagen type I*;

d *human amniotic mesenchymal stromal cells*;

e *imidazole groups-modified gelatin methacrylate*;

f *horseradish peroxidase/glucose oxidase*;

g *Gelatin-Hydroxyphenyl*;

h *human umbilical cord mesenchymal stem cells*;

i *hyaluronic acid/sodium alginate*;

j *human neural stem/progenitor cells*;

k *human adipose-derived stromal/stem cells*;

l *the neural stem/progenitor cells*;

m *semaphorin 3A*;

n *neural stem cells*;

o *fibroblast growth factor 2*;

p *brain-derived neurotrophic factor*;

q *CXC chemokine receptor 4*;

r *a self-assembling biocompatible peptide*;

s *endothelial progenitor cells*.

One of the difficulties in stem cell therapy is that the stem cell's growth, proliferation, and differentiation require a suitable microenvironment. But there are great differences in the microenvironment of tissues in the human body. Under pathological conditions, the damaged area accumulates substances unfavorable for the survival of stem cells. Therefore, the survival rate of implanted stem cells has been unsatisfactory for a long time. As mentioned earlier, as a synthetic material, hydrogels have strong plasticity. With the continuous development of material science, we can now build hydrogels with materials with different strengths and stiffness according to tissue specificity. As the scaffold for stem cells, we can change the mesh size and porosity of hydrogels to adapt stem cells in different sizes. The porous structure is also conducive to the exchange of nutrients, oxygen, and carbon dioxide and the emission of metabolites, providing a friendly environment for the survival, proliferation, and diffusion of stem cells (58). Yao et al. believe stem cell scaffolds should have a series of basic features that support the growth of transplanted cells and match the microenvironment of brain tissue, including rapid gelation process, high water content, porosity, and appropriate rheological behavior and degradation properties. The injectable hydrogel synthesized in this way will have low immunogenicity and cause minimal inflammation *in vivo*. For instance, the gelatin-hydroxyphenyl hydrogel of GO_0.1U_HRP_0.5U_ has sufficient moisture (more than 90%) and appropriate rheological properties (100–1,000 Pa), meeting the physiological characteristics of brain tissue and reducing frictional stimulation to surrounding tissues. Proper gelation time (6 min) also avoids the loss of stem cells caused by longer gelation progress (56). Zhang et al. point out that the rapid flow of cerebrospinal fluid, the mechanical injury, and the reactive inflammation at the lesion induce the high loss rate and low survival rate of transplanted cells (57). Hydrogels mimic the function of the extracellular matrix. The porous structure and suitable pore size improve the survival rate of stem cells and affect the behavior of transplanted cells, such as proliferation and differentiation, promoting the growth of stem cells. Moreover, Xue et al. have tried co-transplantation of neural stem cells and endothelial progenitor cells. Results showed that revascularization, tissue repair, and generation of neural cells were all improved (62).

Another advantage of hydrogels in stem cell therapy is that hydrogels can carry with other macromolecular substances or be modified by certain chemical groups during the synthesis process. These macromolecular substances or chemical groups give hydrogels additional functions, supporting the cellular behaviors of stem cells. For example, SDF-1 and its receptor CXCR4 are key molecules for the survival, migration, and differentiation of many stem cells like neural stem cells or mesenchymal stem cells. Therefore, some employed the slow-release property of SDF-1 in the pores of hydrogels to provide a suitable microenvironment for the survival, migration, and differentiation of transplanted cells (53, 55, 61, 63). Immunogenicity is one of the reasons for the low survival rate of transplanted stem cells. Some researchers have made full use of this advantage of hydrogels to reduce immune rejection. Alvarado-Velez et al. designed a FasL-agarose hydrogel. The sustained release of FasL at the injury site induced apoptosis of CD8^+^ T cells, improving the survival rate of mesenchymal stem cells (MSCs). Meanwhile, they demonstrated that the neurotrophic factors NGF and BDNF significantly upregulated when used FasL-agarose hydrogel, indicating a better therapeutic effect promoted by prolonged MSC survival (54). However, some have adopted distinct design ideas. Betancur et al. constructed a CS-GAG hydrogel to maintain the undifferentiated state of neural stem cells within 4 weeks. Undifferentiated stem cells inhibited the proliferation of reactive astrocytes post-TBI through paracrine action (60). Xu et al. constructed a hydrogel containing semaphorin 3A (Sema3A) gradient, inducing substantial migration of endogenous neural progenitor cells into the hydrogel and promoting their differentiation to regenerate cortical tissue (64).

## Exosome

Current research suggests that exosomes as endosome-derived membrane-bound vesicles can be released by cells in all living systems under any conditions (65). Exosomes are composed of uniform lipid bilayer membranes, containing several vital proteins, including CD63, CD8, CD9, and endosomal membrane proteins flotillin and ALIX. The most significant function of exosomes is carrying macromolecular substances such as proteins, lipids, and nucleic acids to participate in intercellular communication. Among the cargo of exosomes, miRNA is the most well-studied. And there is substantial evidence that miRNA is central to the treatment effects of exosomes. Mature miRNAs have a double-stranded structure, one or both of them will bind to argonaute 2 (Ago2) and be integrated into the RNA-induced silencing complex to cleave the target mRNA or inhibit translational repression (66–68).

Several types of neural cells release exosomes. The glutamatergic synaptic activity in mature neurons promotes the release of exosomes which carry with molecules to modulate neuronal function (65). Exosomes participate in the communication between neurons and endothelial cells, regulating the BBB (59). Oligodendrocytes and astrocytes also communicate with neurons through exosomes (69). The release of exosomes in oligodendrocytes and astrocytes is regulated by the level of cytoplasmic calcium and the concentration of potassium chloride, respectively. And glutamate released by neurons increases exosomes production in oligodendrocytes (70, 71). The exosomes containing the myelin proteins from oligodendrocytes are often around axons, coordinating axon myelination (70, 72, 73). Moreover, oligodendrocyte exosomes transfer superoxide dismutase and catalase to neurons to increase the vitality of neurons under hypoxia-glucose deprivation (OGD) conditions (72). There is evidence indicating that the extensive action of astrocytes depends on the exosomes. The exosomes carrying miR-26 released by astrocytes may inhibit glycogen synthase kinase 3β, an effective inhibitor of axon remodeling and synaptic plasticity (74, 75). In addition, some evidence suggests that astrocytes exosomes transfer prion protein (PrP) to hypoxia and glucose deprivation neurons to prevent neuronal death (76).

It was recorded for the first time that intravenous injection of MSC-derived exosomes in rodent models with TBI significantly increased neurovascular remodeling at the site of the lesion, improving the neurological, behavioral, and cognitive outcomes. The therapeutic gain is equivalent to MSC therapy (77–79). Immunogenicity or histocompatibility also should be considered for exosome therapy. Although there is currently limited data reporting noticeable adverse immune reactions (65).

Exosome therapy is currently commonly administered *via* intravenous or nasal. Many related studies support that under the existing route of administration, exosomes need to cross the BBB to interact with target cells in brain tissue (80–84). Systemic administration of exosomes may promote endogenous neural circuitry reconstruction, white matter remodeling, oligodendrogenesis, angiogenesis, and neurogenesis to improve neurological outcomes. *In vitro* studies show that exosomes derived from MSCs or fibroblasts directly strengthen the outgrowth of dendrites and axons (85, 86). The exosomes from brain endothelial cells of rodents and humans and neural stem cells of adult rodents have been observed promoting neurogenesis and angiogenesis in the process of injury recovery (87–89). In addition to directly affecting nerve repair, exosomes also have indirect effects. In a cerebral ischemia rat model, exogenous exosomes from MSC stimulate astrocytes to release endogenous exosomes assisting exogenous exosomes to promote the growth of cortical neurons (90). Many studies have indicated that the intercellular communication of exosomes participated in the inflammation after brain injury (91–93). And the neuroinflammation was downregulated by injecting MSC-derived exosomes (94, 95). The preclinical study of the TBI model receiving exosome therapy also describes the improved neurological function during the recovery period (92). Moreover, exosomes transfer microglia/macrophages toward anti-inflammatory phenotypes directly by macromolecular substances, such as miRNAs and proteins, or indirectly by upregulating pro-inflammatory factors, such as TNF-α and IL-1β (83, 86, 89, 90, 96).

The main challenge of TBI exosome therapy currently is to develop exosomes targeting specific cells in the central nervous system more effectively than cell-derived exosomes. The application of virus-derived peptides makes it possible for exosomes to target specific cells. Exosomes expressing virus-derived peptides can span the BBB, binding to the corresponding receptors on the surface of cells and importing molecules such as miRNA and siRNA (97–99). However, the virus-derived peptides perhaps induce immune response (100). Studies have shown that exosomes modified by chemically synthesized peptides could avoid immunogenicity (101, 102). Apparently, due to the capacity of carrying and editing, treatment methods based on exosomes have unlimited possibilities.

## Application Prospects of Hydrogel With Exosomes in TBI Therapies

Although some studies have proved that exosomes take effects by crossing the BBB and targeting specific cells, there is evidence suggesting that the existing methods of administration fail to improve the prognosis of TBI (103). An early study finished by Harting et al. using intravenous injection of BMSC-derived exosomes indicated that only a few exosomes would reach the brain (104). In addition, under the traditional route of administration, exosomes have short duration of action and require frequent administration to achieve therapeutic effects, limiting clinical transformation. Because of these limitations, it is worthy to improve the delivery method of exosomes.

As we reviewed above, hydrogels have been extensively studied as scaffolds for stem cell therapy of TBI, obtaining many positive results. And the method of encapsulating exosomes with hydrogels has been applied in several fields such as bone and cartilage regeneration (105, 106), diabetic chronic wound healing (107, 108), and cardiac repair (109, 110). Moreover, some research on spinal cord injury (SCI) repair attempting to embed exosomes into injectable hydrogels confirmed the effective retention and slow release of exosomes at the target site, with enhanced angiogenesis and neural function (111–114).

Here, we summarize the possible advantages of hydrogels for exosome treatment in TBI: 1. Direct access to the lesion. Compared to the application of exosomes *via* intravenous or nasal routes, *in situ* injections of hydrogels can directly reach the lesion and act at the injured site, avoiding the influence of BBB, and improving the treatment efficiency of exosomes. 2. Filling the cyst cavity. The formation of the cystic cavity post-TBI is quite common, unfavorable for nerve repair and regeneration at the injured site. It is difficult to change this with the traditional route of administration of exosomes, while hydrogels can perfectly fill the cyst cavity and provide a better microenvironment for nerve regeneration. 3. Editability. As a synthetic material, hydrogels have excellent editability. Its pore size can be controlled to adapt to varied sizes of exosomes by selecting various materials and changing synthesis conditions. In addition, it is foreseeable that by selecting materials with different characteristics or adding different chemical group modifications, hydrogels can also obtain additional capacities, such as regulating the release conditions and release curves of exosomes and creating a favorable microenvironment. Recently, Staufer et al. developed a fully synthetic extracellular vesicle and demonstrated its therapeutic function, remarking a new level of editability of exosomes (115). In the future, by combining the two highly programmable tools of hydrogels and exosomes, we have great reason to believe that the treatment of TBI will usher in the dawn.

Of course, there are also foreseeable drawbacks in therapy of using hydrogels and exosomes. 1. Although the advantage of hydrogels in the treatment of brain trauma is to fill the cavity formed after trauma and directly contact the lesion, the accompanying side effect of hydrogels may be the mechanical damage to the surrounding brain tissue. Compared with the traditional covalent gel, the dynamic hydrogel is injectable and gelling *in situ*, substantially reducing mechanical damage during implantation. However, injectable hydrogels are not perfect. These hydrogels usually experience problems with gelation kinetics, such as coagulating too quickly in the syringe, gelling so slow that the drugs release prematurely, or heterogeneous gelation caused by poor mixing. Therefore, dynamic hydrogels capable of seamlessly transition between solid-like and liquid-like during injection draw much attention (6). 2. Risks of the implant. Most hydrogel matrices used recently are synthetic materials, reducing the risk of rejection after implantation compared with bio-derived materials. However, risks of hydrogels still exist objectively, such as immune response and infection. As for exosomes, immune reaction may also be a disadvantage of our concern, as described above in this review. 3. In addition, injectable hydrogels still have problems to be solved before clinical trials. For example, most researchers directly left empty cavities in TBI animal models, injecting hydrogels into them. Although they present positive results, it only illustrates that injectable hydrogels are suitable for TBI patients who need surgery, which undoubtedly restricts the application of hydrogels. However, the majority of TBI patients only take conservative treatment in clinical. For such patients, the validity and safety of injectable hydrogels need more vivo trials.

Although the injectable hydrogel reduces mechanical damage during implantation compared with the traditional gel, the possibility of mechanical damage still exists objectively. We believe that this damage is due to the material properties of hydrogels. Some characteristics can help us evaluate and reduce the mechanical damage. 1. The mechanical strength of hydrogels. Matthew reveals that if the storage moduli of the hydrogel similar to the brain (140–620 Pa), the hydrogel can maintain tight apposition to brain tissue (55). In addition, to prevent it from being squeezed out by the surrounding tissue, the deformation resistance of the hydrogel can be adjusted by the chemically modified matrix. Yao et al. also proposes that the hydrogel possesses high water content (>90%) and appropriate rheological behavior (100–1,000 Pa) can reduce the frictional irritation to the surrounding tissue (56). 2. Swelling behavior. When certain materials such as HA are used for hydrogel preparation, the hydrogel shows water intake capacity. The rapid and high swelling behavior may stress the surrounding tissues, causing secondary mechanical damage (116). However, we can choose a different material ratio to synthesize hydrogels with appropriate swelling ability to reduce this type of damage.

Based on the experience of hydrogels with drugs or stem cell therapies before, the ideal characteristics for the hydrogel include the following: 1. Be injectable and be able to gel *in situ*. 2. Mechanical properties comparable to brain tissue to minimize mechanical injury. 3. Be biocompatible and biodegradable to reduce risks of immune response and support tissue regeneration. 4. Be made of a material that appropriate mesh size allows sustained release of exosomes (117).

## Conclusion

We briefly review the pathophysiological mechanism of TBI and describe the potential new treatment strategies for TBI. The therapies for TBI are problematic due to the modes of drug or cell delivery. Hydrogels have the potential of conveying drugs or cells to the target injured sites and improving their effects. The therapeutic gain of exosome therapy may be equivalent to stem cell therapy. And the low immunogenicity of exosomes suggests that the adverse effects associated with current stem cell therapies can be reduced. Evidence from studies regarding the employment of exosomes and hydrogels co-therapies in TBI are lacking. Although we believe co-therapies employing exosomes and hydrogels can potentially ameliorate TBI, it still needs to be confirmed by thorough multitudinous studies in the future.

## Author Contributions

YC, JL, and WY: conceptualization. YC: writing—original draft preparation. JL and WY: writing—review and editing. All authors have read and agreed to the published version of the manuscript.

## Conflict of Interest

The authors declare that the research was conducted in the absence of any commercial or financial relationships that could be construed as a potential conflict of interest.

## Publisher's Note

All claims expressed in this article are solely those of the authors and do not necessarily represent those of their affiliated organizations, or those of the publisher, the editors and the reviewers. Any product that may be evaluated in this article, or claim that may be made by its manufacturer, is not guaranteed or endorsed by the publisher.
