# Corrigendum: A prosperous application of hydrogels with extracellular vesicles release for traumatic brain injury

**DOI:** 10.3389/fneur.2022.984818

**Published:** 2022-08-03

**Authors:** Yang Chen, Jingquan Lin, Wei Yan

**Affiliations:** Department of Neurosurgery, The Second Affiliated Hospital, Zhejiang University School of Medicine, Hangzhou, China

**Keywords:** hydrogels, exosomes, traumatic brain injury, biocompatible materials, therapy

In the published article, an author name was incorrectly written as “Jinquan Lin.” The correct spelling is “Jingquan Lin.”

The authors apologize for this error and state that this does not change the scientific conclusions of the article in any way. The original article has been updated.

## Publisher's note

All claims expressed in this article are solely those of the authors and do not necessarily represent those of their affiliated organizations, or those of the publisher, the editors and the reviewers. Any product that may be evaluated in this article, or claim that may be made by its manufacturer, is not guaranteed or endorsed by the publisher.

